# The Frontiers of Madness

**Published:** 1883-03

**Authors:** 


### THE FRONTIERS OF MADNESS.

"The Frontiers of Madness" is the title of an interesting lecture recently delivered by Dr. Ball, in his course at the Paris Faculty of Medicine. The generally received opinion that folly and reason are separated by a strictly-drawn mathematical line, is, according to Dr. Ball, quite erroneous. There is a broad frontier, he says, between sanity and insanity, which is peopled by millions of inhabitants. Damasippus, in Horace, laid down the doctrine that all men are mad—"*insanus et tu, stultique prope omnes.*" Dr. Ball, without going quite so far as this, holds that the number of persons perfectly reasonable on all points throughout the entire period of their existence, form but a minority of mankind. The world abounds with people, he tells us, whom a strict scientific diagnosis would condemn as mad, or more or less "touched;" yet at no time of their life would it be permissible to put them under restraint. Such persons are to be seen occupying honorably and successfully every position in life and society. We brush against them when we take our daily walks abroad; we see them in the mirror which reflects ourselves.

Dr. Ball, having stated the thesis of his discourse, proceeds to a classification of these "sane madmen," and designs the first place "in the order of merit" (from what point of view he does not specify) to those who suffer from unreasonable, aud, in most cases, irresistible impulses. Naturally enough, the lecturer referred to the case of Dr. Johnson, and the curious impulse which prompted him to touch each post as he walked along the streets —an impulse so strong that if he accidentally passed one by without the usual tribute of a touch, he felt irresistibly compelled to return and repair the omission. The overpowering impulse to laugh on occasions of peculiar solemnity is one which even the most serious persons have experienced. A still more morbid impulse is that which sometimes urges pious people to indulge in blasphemous or profane language. A great English divine, Bishop Butler, was tormented all his life long by this temptation, which he only mastered by strong and sustained efforts of the will. The impulse sometimes assumes a suicidal form.

Dr. Ball was recently visited by a young man who was engaged to be married, but who found it impossible to visit his intended bride, because it would involve a journey of some length in a railway carriage, and he could never enter one without feeling a desire to jump out as soon as the train was in motion. He was advised to accustom himself gradually to this mode of traveling by taking short journeys on the suburban line, but he could never get beyond Auteuil; there he had to leave the carriage for fear of accident. Thouviot's case is one of the oftenest quoted. For years this unpleasant person was tortured with a burning desire to kill some woman or other; but he never felt the slightest wish to take the life of a man. He battled with the impulse for years, but at last it got the better of him. One day he murdered a young girl, a perfect stranger to him, whom unfortunate chance threw in his way in the kitchen of a restaurant. Dr. Ball was consulted some time ago by a painter of considerable talent, who was a prey to these murderous impulses. He had married early in life; his family was large, and his cares and anxieties large in proportion. At about thirty-eight, without physical ailment of any kind, or any specially unfavorable turn in his affairs, his mind began to be affected. If he saw a mirror, he experienced a desire to smash it; near a window, he felt a temptation to jump out; he never got a bank-note into his hand that he did not feel impelled to tear it in pieces. These morbid promptings presently assumed a more formidable shape; he began to be assailed with a temptation to strangle his children. His little daughter was dying of croup, and he spent night after night by her bedside, nursing her with the utmost tenderness. "Yet, said he to the physician, "at the moment when I was praying, with tears in my eyes, that the child's life might be spared, I was tormented with a horrible desire to take her out of the cradle and throw her into the fire. Even now," he added, "as I speak to you, I feel a most intense desire to strangle you; but I check myself."

It is a relief to turn from this tragic side of the subject to the comic aspects which it presents in the impulses of kleptomaniacs. The disposition to purloin objects of little or no value often manifests itself in persons of wealth and position quite removed from the reach of vulgar temptations. Some kleptomaniacs will only take objects of a particular sort. Peddie mentions the case of a very pious person who stole all the bibles he could lay his hands on. Another kleptomaniac only stole clothes-pegs, and, as he had absolutely no use for them, he amassed at last quite a collection. Another worthy man's respect for the distinction of *meum* and *tuum* broke down at once before a blue cotton nightcap, although he never wore any but white nightcaps himself.

The tyranny of a fixed idea is another form which the madness of some people assumes. One sufferer feels an unctuous sensation all over his body, and takes it into his head that he has been dipped in grease. Another, a studious, intelligent young man, is obliged to give up reading altogether, because each time he turns over a page he imagines he has skipped a leaf. Back he is obliged to go again, again the fancy returns, and so he never makes progress. Dr. Cabade had once a patient whom he described as an excellent man of business, who nevertheless felt himself unable, though free from the slightest physical weakness, to perform some of the simplest acts of daily life. He could not cross the threshold of his door without being pushed from behind. He could not rise from his chair without calling for help. In the street his progress was liable to be stopped at any moment by some imaginary obstacle, which no effort of the will would enable him to cross. Akin to these cases, and not readily distinguishable from some of them, is that of the class of persons whom we term hypochondriacs. Every one numbers among his acquaintances some one who never tires of talking of the imaginary ailments from which he is suffering. Many a medical student has been driven half crazy from fancying that he had himself the symptoms of the different diseases which his books describes. Perfectly sane people, again, suffer from hallucinations of one sort or other. Lelorgne de Savigny, for instance, to quote a well-known case, was afflicted with visual hallucination, of which he has left us an account; and which were of so painful a character that he at last shut himself in a perfectly dark room, and passed the remainder of his days there, having failed of obtaining relief in any other way. And there is the case of a drunkard who labored under a curious hallucination of the faculty of hearing.

He rose every morning full of the best resolutions, and determined to keep sober for the day. Unfortunately, the road to where he worked, passed by a certain public house, and at some distance from this fatal spot he became conscious of two voices crying in his ear, the one "He will not go in," the other, "He will go in." As he got near the door the voice of the tempter increased in force till it quite drowned that of the good angel. The matter always ended by his going in and taking a drink, when the hallucination ceased as if by enchantment.—*St. James's Gazette.*

